# Reply to Possenriede et al.: LDLR as a targetable HlyA receptor in *Escherichia coli* pyelonephritis

**DOI:** 10.1073/pnas.2522220122

**Published:** 2025-09-15

**Authors:** Hunter W. Kuhn, Madeleine R. Smither, Christina A. Collins, Joseph P. Gaut, Michael S. Diamond, David A. Hunstad

**Affiliations:** ^a^Department of Pediatrics, Washington University School of Medicine, St. Louis, MO 63110; ^b^Department of Pathology & Immunology, Washington University School of Medicine, St. Louis, MO 63110; ^c^Department of Medicine, Washington University School of Medicine, St. Louis, MO 63110; ^d^Department of Molecular Microbiology, Washington University School of Medicine, St. Louis, MO 63110

We read with interest the letter and data shared by Possenriede et al. (1) in response to our recent *PNAS* publication (2) identifying the low-density lipoprotein receptor (LDLR) as mediating the toxicity of *Escherichia coli* alpha-hemolysin (HlyA) toward renal epithelial cells.

In experimental pyelonephritis with uropathogenic *E. coli* (UPEC) strain 536, these investigators found statistically larger renal scar area, as measured by trichrome stain positivity, in wild-type B6 mice compared to *Ldlr^–/–^* mice 11 wk after infection (1). They observed no difference in kidney bacterial loads between host genotypes, aligned with observations by us and others (3) that HlyA expression by *E. coli* does not affect bacterial loads during experimental UTI. We agree that these tantalizing data further support a role for the HlyA–LDLR interaction in driving renal epithelial injury during UPEC pyelonephritis. As the authors point out, comparative experiments with an isogenic 536 Δ*hlyA* mutant would be necessary to specify whether their observation reflects activity of HlyA. In addition, as germline *Ldlr* lack may exert more generalized effects on host metabolism and physiology that might influence infection susceptibility (4), we are pursuing experiments with conditional and inducible *Ldlr* disruption in renal epithelial cell populations along the nephron.

Though a few preclinical UTI studies implicate HlyA in local renal tissue injury and scar formation (3, 5), data supporting this link in human populations are lacking. Several studies have broadly sought host genetic determinants of risk for pyelonephritis. Svanborg and colleagues, in a genome-wide association study (GWAS), identified variants in *CXCR1* (encoding IL-8 receptor) associated with acute pyelonephritis (6, 7). A Japanese cross-population analysis of variant association with over 200 distinct phenotypes (of which pyelonephritis was one) identified a locus on chromosome 14 near *LINC02302* (8). Last year, a GWAS in populations of European ancestry did not replicate the “hits” from these prior studies but did identify a pyelonephritis-associated variant on chromosome 2 near *TSN*, reaching significance only in females (9). Case definitions and subject ascertainment methodologies were not identical among these studies, likely explaining the differing results. Regardless, we would not expect *Ldlr* to have been identified, as the available data indicate HlyA is not necessary for the establishment of pyelonephritis but contributes specifically to renal tissue injury and possibly to scarring. The preliminary analysis of human *LDLR* shared by Possenriede et al. (1) provides strong premise for further interrogation of *LDLR* variation, especially in carefully curated cohorts with *E. coli* pyelonephritis in whom *hlyA* carriage by the infecting strains, and scarring outcomes, are known.

We appreciate these authors’ correspondence and are optimistic about prospects for targeting the HlyA–LDLR interaction to mitigate tissue damage and long-term sequelae in UPEC pyelonephritis.
